# Supplementary material for: Cervicovaginal Microbiota Composition in Chlamydia trachomatis Infection: A Systematic Review and Meta-Analysis
Source: Int J Mol Sci. 2022 Aug 23;23(17):9554. doi: 10.3390/ijms23179554 (PMC9455926; doi:10.3390/ijms23179554)
Supplement: Supplementary file 1 [file ijms-23-09554-s001.zip › ijms-1861523-supplementary.pdf]

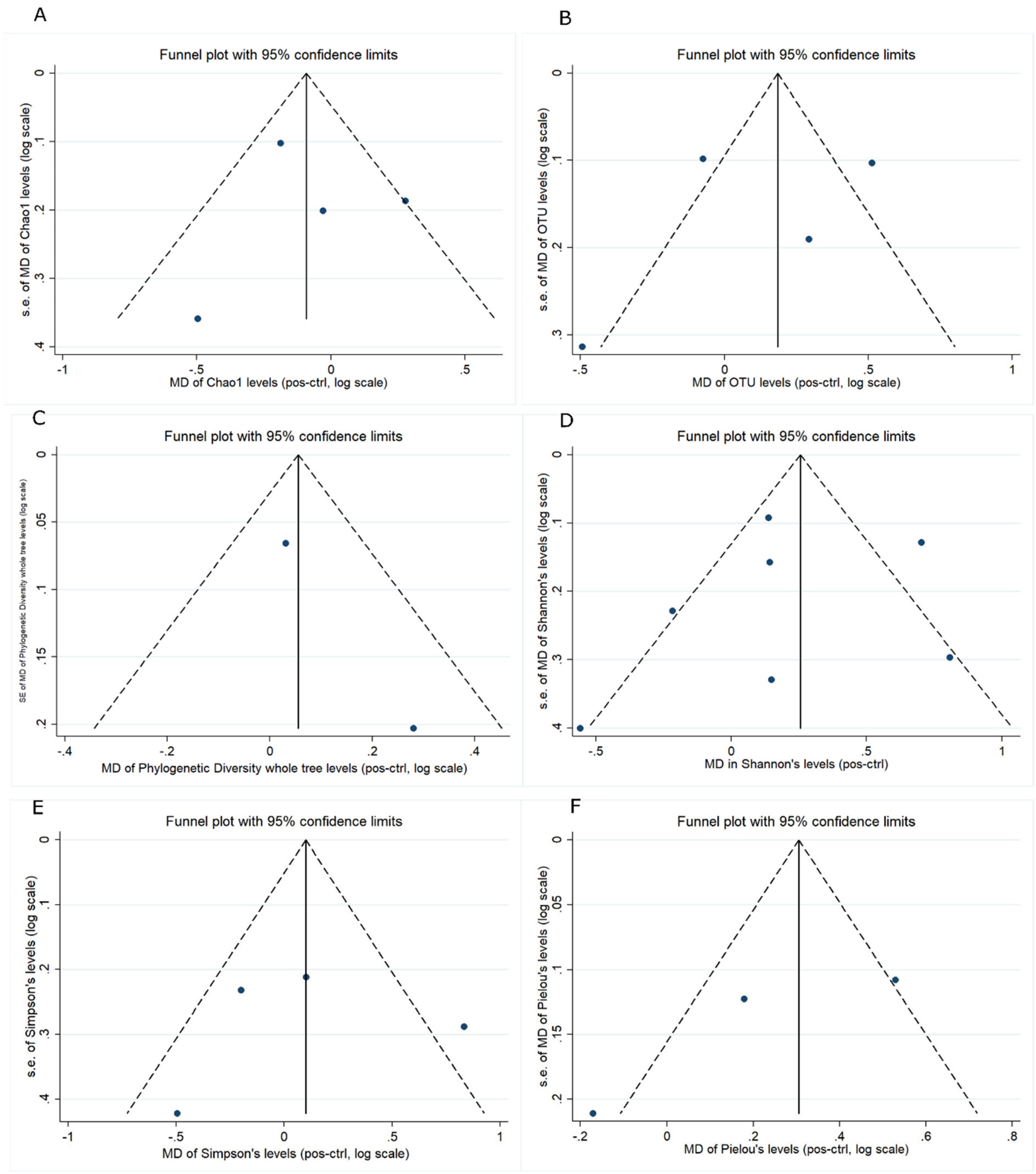

Figure S1. Funnel plots of Standard Error (se) versus the the Mean Difference (MD) on logarithmic scale between positive group (pos) and healthy controls (ctrl) in the levels of each Alpha-Diversity parameters (**A**, Chao1; **B**, OTU number; **C**, Phylogenetic diversity whole tree; **D**, Shannon; **E**, Pielou's evenness; **F**, Simpson). The dots represent point estimate from each study, larger studies will appear at the top of the funnel, while in contrast, effect estimates from smaller studies (studies with higher se so less precision) may be expected to scatter near the bottom of the funnel.

Table S1. Data extracted and collected for each outcome considered in the meta-analyses, MD logarithmic transformation by method 3 of Higgins et al. [33].

**First Author - Publication Year**	***C. trachomatis* positive (positive group)**	***C. trachomatis* negative (healthy controls group)**	**Chao1**						**OTU**						**Phylogenetic Diversity whole tree**					
			**Raw scale**				**logarithmic scale**		**Raw scale**				**logarithmic scale**		**Raw scale**				**logarithmic scale**	
			**Positive group**		**Controls group**		**MD**	**SE**	**Positive group**		**Controls group**		**MD**	**SE**	**Positive group**		**Controls group**		**MD**	**SE**
	**N**	**N**	**Mean**	**SD**	**Mean**	**SD**			**Mean**	**SD**	**Mean**	**SD**			**Mean**	**SD**	**Mean**	**SD**		
**Chen H et al. - 2021**	6	8	468.74	469.55	777.73	327.01	-0.50	0.36	258.33	210.96	427.86	182.55	-0.49	0.31						
**Masha SC et al. - 2019**	14	21	-	-	-	-	-	-	-	-	-	-	-	-	-	-	-	-	-	-
**Cheong H et al. - 2019**	42	35	-	-	-	-	-	-	-	-	-	-	-	-	-	-	-	-	-	-
**Ceccarani C et al. - 2019**	20	21	6618.54	1599.84	7980.26	3015.39	-0.19	0.10	1276.25	324.45	1374.95	495.89	-0.07	0.10	71.77	8.55	69.53	19.42	0.03	0.07
**Filardo S et al. - 2019**	39	99	-	-	-	-	-	-	16.47	7.84	9.75	4.99	0.51	0.10	-	-	-	-	-	-
**Filardo S et al. - 2017**	7	7	12.99	3.48	9.81	4.44	0.28	0.19	12.56	3.65	9.34	4.14	0.29	0.19	3.77	1.00	2.84	1.47	0.28	0.20
**Raimondi S et al. - 2021**	10	16	95.23	46.11	98.04	51.70	-0.03	0.20	-	-	-	-	-	-	-	-	-	-	-	-

**First Author - Publication Year**	***C. trachomatis* positive (positive group)**	***C. trachomatis* negative (healthy controls group)**	**Shannon's**						**Pielou's**						**Simpson's**					
			**Raw scale**				**logarithmic scale**		**Raw scale**				**logarithmic scale**		**Raw scale**				**logarithmic scale**	
			**Positive group**		**Controls group**		**MD**	**SE**	**Positive group**		**Controls group**		**MD**	**SE**	**Positive group**		**Controls group**		**MD**	**SE**
	N	N	**Mean**	**SD**	**Mean**	**SD**			**Mean**	**SD**	**Mean**	**SD**			**Mean**	**SD**	**Mean**	**SD**		
**Chen H et al. - 2021**	6	8	1.32	1.29	2.34	1.44	-0.56	0.40	-	-	-	-	-	-	0.32	0.36	0.53	0.29	-0.49	0.42
**Masha SC et al. - 2019**	14	21	1.95	1.27	1.68	2.25	0.15	0.33	-	-	-	-	-	-	0.75	0.30	0.68	0.59	0.10	0.21
**Cheong H et al. - 2019**	42	35	0.43	0.44	0.53	0.51	-0.22	0.23	0.15	0.15	0.18	0.16	-0.17	0.21	0.22	0.23	0.27	0.26	-0.20	0.23
**Ceccarani C et al. - 2019**	20	21	4.03	0.89	3.51	1.31	0.14	0.09	-	-	-	-	-	-	-	-	-	-	-	-
**Filardo S et al. - 2019**	39	99	1.51	0.81	0.72	0.63	0.70	0.13	0.37	0.17	0.22	0.18	0.53	0.11	-	-	-	-	-	-
**Filardo S et al. - 2017**	7	7	1.46	0.66	0.62	0.47	0.81	0.30	-	-	-	-	-	-	0.51	0.21	0.21	0.18	0.83	0.29
**Raimondi S et al. - 2021**	10	16	4.10	1.33	3.55	1.73	0.14	0.16	0.64	0.15	0.54	0.22	0.18	0.12	-	-	-	-	-	-
